# Factors associated with the difference between the incidence and case-fatality ratio of coronavirus disease 2019 by country

**DOI:** 10.1038/s41598-021-98378-x

**Published:** 2021-09-23

**Authors:** Jeehyun Kim, Kwan Hong, Sujin Yum, Raquel Elizabeth Gómez Gómez, Jieun Jang, Sun Hee Park, Young June Choe, Sukhyun Ryu, Dae Won Park, Young Seok Lee, Heeyoung Lee, Dong Hyun Kim, Dong-Hyun Kim, Byung Chul Chun

**Affiliations:** 1 Department of Preventive Medicine, Korea University College of Medicine, Seoul, Republic of Korea; 2 Graduate School of Public Health, Korea University, Seoul, Republic of Korea; 3 Transdisciplinary Major in Learning Health Systems, Department of Healthcare Sciences, Graduate School, Korea University, Seoul, Republic of Korea; 4 Division of Infectious Diseases, Department of Internal Medicine, College of Medicine, The Catholic University of Korea, Seoul, Republic of Korea; 5 Department of Pediatrics, Korea University Anam Hospital, Seoul, Republic of Korea; 6 Department of Preventive Medicine, Konyang University College of Medicine, Daejeon, Republic of Korea; 7 Division of Infectious Diseases, Department of Internal Medicine, Korea University Ansan Hospital, Ansan, Republic of Korea; 8 Division of Pulmonary, Allergy, and Critical Care Medicine, Department of Internal Medicine, Korea University Guro Hospital, Seoul, Republic of Korea; 9 Center for Preventive Medicine and Public Health, Seoul National University Bundang Hospital, Seongnam, Republic of Korea; 10 Department of Pediatrics, Inha University School of Medicine, Incheon, Republic of Korea; 11 Department of Social and Preventive Medicine, Hallym University College of Medicine, Chuncheon, Gangwon, Republic of Korea

**Keywords:** Diseases, Health care, Medical research, Risk factors

## Abstract

Coronavirus disease (COVID-19) has been spreading all over the world; however, its incidence and case-fatality ratio differ greatly between countries and between continents. We investigated factors associated with international variation in COVID-19 incidence and case-fatality ratio (CFR) across 107 northern hemisphere countries, using publicly available COVID-19 outcome data as of 14 September 2020. We included country-specific geographic, demographic, socio-economic features, global health security index (GHSI), healthcare capacity, and major health behavior indexes in multivariate models to explain this variation. Multiple linear regression highlighted that incidence was associated with ethnic region (*p* < 0.05), global health security index 4 (GHSI4) (beta coefficient [β] 0.50, 95% Confidence Interval [CI] 0.14–0.87), population density (β 0.35, 95% CI 0.10–0.60), and water safety level (β 0.51, 95% CI 0.19–0.84). The CFR was associated with ethnic region (*p* < 0.05), GHSI4 (β 0.53, 95% CI 0.14–0.92), proportion of population over 65 (β 0.71, 95% CI 0.19–1.24), international tourism receipt level (β − 0.23, 95% CI − 0.43 to − 0.03), and the number of physicians (β − 0.37, 95% CI − 0.69 to − 0.06). Ethnic region was the most influential factor for both COVID-19 incidence (partial ${R}^{2}$ = 0.545) and CFR (partial ${R}^{2}$ = 0.372), even after adjusting for various confounding factors.

## Introduction

Coronavirus disease (COVID-19), caused by the novel severe acute respiratory syndrome coronavirus 2 (SARS-CoV-2), was declared as pandemic by World Health Organization (WHO) on 11 March 2020. Until the end of July 2021, pandemic has resulted in 196 million confirmed cases and more than four million deaths worldwide^1^. There is variation in the caseloads and severities of COVID-19 across continents and countries^1,2^. The Americas reported confirmed cases per one million population approximately 52.7 times higher confirmed cases per one million population than that of Western Pacific as of 16 August 2020 and these regional differences are not vanished even as the pandemic continues, Americas being approximately 33.3 times higher than Western Pacific as of 31 July 2021^1^. The reason of the difference should be investigated, considering Western Pacific, where the disease occurred at first, had notably lower incidence and mortality than the Americas and Europe, where most of the industrialized countries with sufficient healthcare capacity and hygiene condition are.

Numerous studies investigating the risk factors for COVID-19 outcomes within countries have been published. The examined risk factors are demographic features, comorbidities, socioeconomic disparity, and environmental features^3,4^ at the district level. Specifically, male sex^5–7^, older age^7,8^, comorbidities^6,7,9–11^ are suggested as factors that increase the risk of negative COVID-19 outcomes. Socioeconomic disparities, such as income level^12–14^, education^12,13^, and unemployment^14^, are reported to be associated with COVID-19. Moreover, ethnicity is suggested to be associated with the disparity of COVID-19 outcomes, although it is not verified whether the underlying cause of the disparity results from biological or socio-economic features of the different ethnicities^8,12,15^. However, there are few country-level studies investigating the possible factors for international variation in COVID-19 outcomes. Clarifying the potential country-level factors could provide evidence for policy makers to implement appropriate COVID-19 control measures, such as social distancing and lockdowns.

Using publicly available data, this study aims to identify the factors related to the international variation of COVID-19 outcomes at the country level and to measure how much each factor could explain the disease outcome, by adjusting for the national COVID-19 test rate, and the demographic and economic features.

## Methods

### Data extraction

We obtained the data on COVID-19 outcomes of each country, i.e., total confirmed cases, recovered cases, deaths, and number of tests performed from Worldometer Coronavirus statistics websites^16^, one of the most popular COVID-19 data sources, at 14 September 2020. We retrieved data at 14 September 2020 so that we could consider that most of the countries had gone through the first wave of COVID-19^17,18^ and chances of biased results caused by possible cocirculation of flu and COVID-19^18^ could be reduced. The number of total confirmed cases per million population was used as COVID-19 incidence and the number of total deaths divided by the number of confirmed cases was used as the CFR (%). Only countries in northern hemisphere were included since northern and southern hemispheres had different prevalence duration of COVID-19, and each hemisphere had different seasonality as of 14 September 2020^4^.

Information on country-level indices, namely, geographic, demographic, and socio-economic features, global health security index (GHSI), healthcare capacity, and health behaviors, were examined for possible factors, considering the results of previous studies which investigated association between COVID-19 health outcomes and each variable^2–15,19–23^. Specifically, information on ethnic region^24^, proportion of female (%)^25^, land area (km^2^)^25^, median age^26^, population over 65 years of age (% of total population)^25^, total population^27^, population density (P/km^2^)^27^, urban population (% of total population)^27^, education index^26^, GDP per capita (current US$)^25^, Gini index^25^ for detection of income dispersion, international tourism receipts (% of total exports)^25^, and unemployment (% of total labor force)^25^ was included in this study. Ethnic region was based on the data of ethnic categories extracted from a previously published article^24^, because recognized social standards that defined ethnic categories at the national level was absent^28^. Rawshani et al.^24^ categorized ethnicity by considering geographical adjoins and evaluating each country’s ethnic composition, economic development, history, and religion. The ethnic region in our research consisted of nine categories: East Asia; Europe (high income), North America and Oceania; Europe (low income), Russia and Central Asia; Latin America and the Caribbean; Mediterranean Basin; Middle East and North Africa; Nordic countries; South Asia; and Sub-Saharan Africa. GHSI was a comprehensive assessment of the health secure capability of a country to prevent and combat epidemic. The index had an overall score and comprised six categories: prevention of pathogen release (GHSI1); detection and reporting for epidemics (GHSI2); rapid response to an epidemic (GHSI3); capability of the health system to treat patients and protect healthcare workers (GHSI4); compliance with international commitments (GHSI5); and, nationwide environmental risk and public health vulnerability to biological threats (GHSI6)^29^. Each category of the GHSI and the overall scores ranged from 0 to 100, with higher scores indicating better preparedness in the corresponding category.

We collected information on healthcare capacity, such as healthcare access and quality (HAQ) index^30^, health expenditure (% of GDP)^25^, out-of-pocket expenditure (% of current health expenditure)^25^, and the number of hospital beds, nurses, and physicians per 1000 people^25^. The HAQ index analyzed the 32 causes of death that are considered avoidable in the availability of quality medical services^30^. Causes of death included various health service areas, such as vaccine-preventable diseases; epidemics and maternal and child health; non-infectious diseases; and, gastrointestinal diseases in which death is preventable by surgery^30^. The values ranged from 0 to 100, and higher values indicate that the country has a higher quality of and better accessibility to medical care^30^. Information on comorbidities and health behaviors which can contribute to COVID-19 outcomes was extracted. We included information on obesity prevalence^31^, diabetes prevalence^25^, smoking prevalence^31^, alcohol consumption^25^, and water, sanitation, and hygiene (WASH) index^32^. The WASH index assesses the safety and accessibility to water and sanitation facilities and personal hygiene levels. The indicators are independent but also interdependent. The values ranged from 0 to 100, with higher scores indicating better conditions for the corresponding factor^32^. The WHO argued that ensuring proper condition of WASH in communities, homes, schools, and medical facilities would help prevent COVID-19 transmission^33^. The WASH index that assessed personal hygiene was excluded from our analysis due to an abundance of missing values (81, 59.6%). There was no duplication between variables. All the data used in this study were publicly available.

### Statistical analysis

The analysis was conducted in country level. Baseline information of variables was assessed with median, mean, minimum, maximum, 25th and 75th percentile. Medians was used for the imputation of missing values of independent variables as the independent variables were not normally distributed.

Multiple linear regression was used to identify potential factors associated with incidence and CFR. Outcome variables, including incidence and CFR, were log transformed for the multiple linear regression analysis. The zero value in the CFR (%) was imputed with 0.005 for log transformation (corresponding country: Laos, Mongolia, and Cambodia). The continuous independent variables were standardized to properly compare the effects of potential factors, as the scale of each factor was different.

Potential predictors were first identified by univariate linear regression with *p* < 0.25 (Tables S2 and S3 in Supplement). A backward elimination was implemented. Then, incidence model embedded variables that stood for sex, age, GDP per capita, and COVID-19 test rates, to verify the effects of potential factors on the disease even after adjusting for the national demographic and economic features, and COVID-19 test rates. CFR model embedded COVID-19 incidence instead of COVID-19 test rates, considering incidence could affect mortality by bringing burden to national capacity against COVID-19 and medical system. Multicollinearity was considered (variance inflation factors (VIF) < 10) for the variable selection. Thus, variables with VIF ≥ 10 were excluded for the final model. The outcomes were presented with beta coefficients (β), 95% confidence interval (CI) of beta coefficients, and partial R-squared statistics. Partial R-squared statistics implicated the explanation portion of each variable in the model. The explanatory power of the model was assessed using adjusted R-squared statistics.

The sub-analyses on 136 countries, including countries in both northern and southern hemisphere, selected variables as the main analysis did. Multiple linear regression on log transformed incidence (Table S4 in Supplement) and log transformed CFR (Table S5 in Supplement) were conducted. We also performed the sub-analyses by each ethnic region respectively, except Mediterranean Basin (N = 5), Nordic countries (N = 4), and Sub-Saharan Africa (N = 4) region because the number of countries included in corresponding regions was less than 10. By each ethnic region, COVID-19 incidence and CFR were dichotomized with the median value (0: lower incidence [CFR]; 1: higher incidence [CFR]). A backward elimination process was implemented on the model with potential factors as which identified by univariate logistic regression with *p* < 0.25. The model on incidence included variables that stood for sex, age, GDP per capita, and COVID-19 test rates, while the model on CFR embedded COVID-19 incidence instead of COVID-19 test rates. Multicollinearity was considered (VIF < 10) when selecting variables for the final model. Multiple logistic regression was conducted, and the results are suggested in Supplementary Tables S6 and S7.

All statistical analyses were performed using R version 4.0.2 (R foundation for Statistical Computing, https://www.r-project.org). We used QGIS version 3.10.13 (QGIS Development Team, http://qgis.osgeo.org) for mapping. The institutional review board (IRB) of Korea University granted exemption for this study (IRB exemption number: KUIRB-2020-0281-01).

## Results

### Characteristics of total selected countries

**Figure 1:**
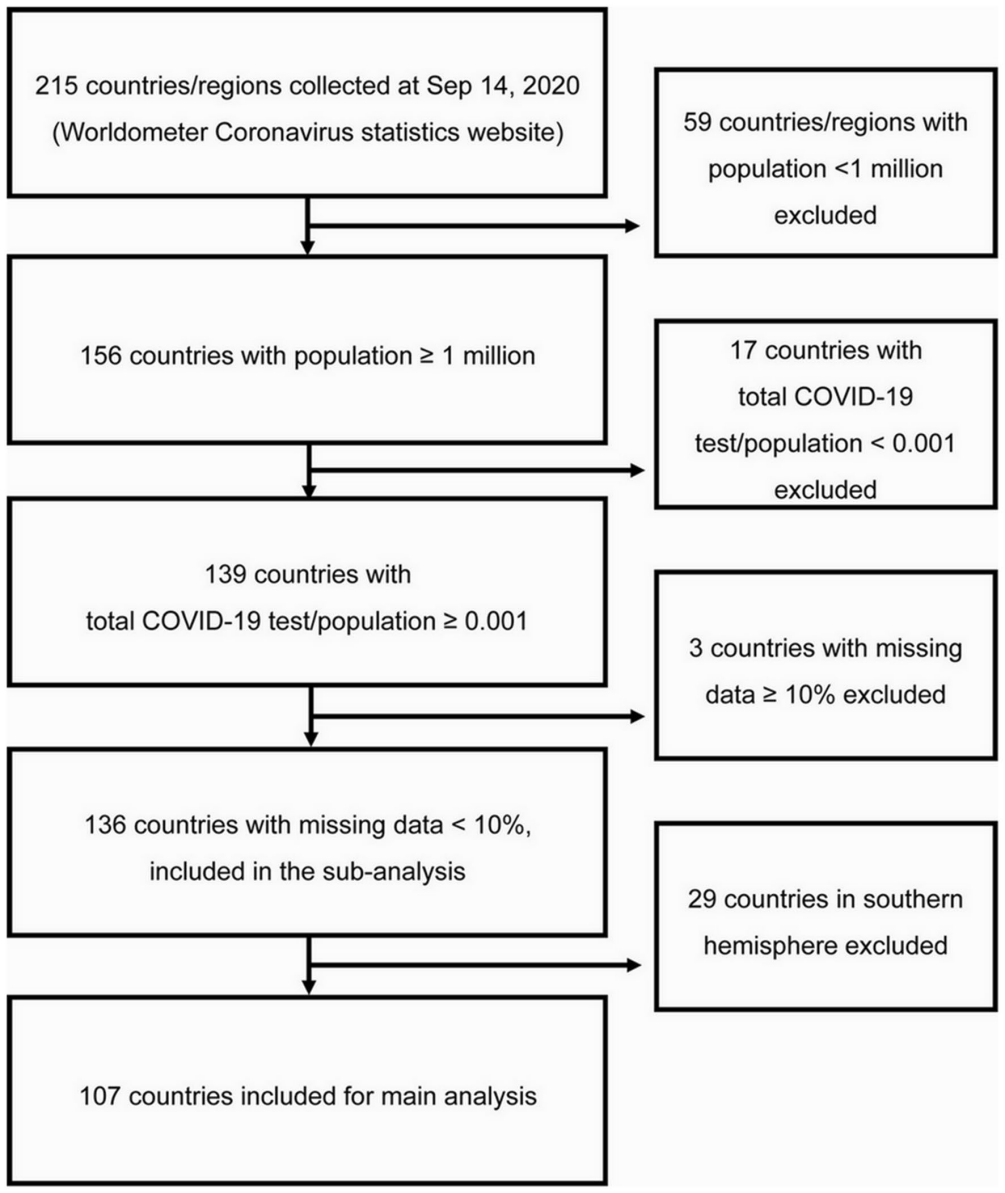
Flowchart of study subject selection. Data was extracted from Worldometer Coronavirus statistics website (https://www.worldometers.info/coronavirus) on September 14, 2020.

There were 215 countries or regions reported on Worldometer site on 14 September 2020. Countries or regions with less than one million population (n = 59), those with lower value than 0.001 for total test per population (n = 17), those with more than 10% missing independent variables (n = 3), and those in the southern hemisphere (n = 29) were excluded. Finally, 107 northern hemisphere countries were included for analysis (Fig. 1). Lists of the countries included in each ethnic region are summarized in Table 1. Among 107 countries, the most frequent ethnic regions were “Middle East and North Africa (21, 19.6%)” whereas “Nordic countries (4, 3.7%)” and “South Asia (4, 3.7%)” were the least frequent.

**Table 1:** Lists of countries in each ethnic region included in this study.

Ethnic region	No. (%)	Countries
East Asia	13 (12.1)	Cambodia, China, Japan, Laos, Malaysia, Mongolia, Myanmar, Nepal, Philippines, Singapore, South Korea, Thailand, Vietnam
Europe (high income), North America & Oceania	20 (18.7)	Austria, Belgium, Bulgaria, Canada, Czechia, Estonia, France, Germany, Hungary, Ireland, Latvia, Lithuania, Netherlands, Poland, Romania, Slovakia, Slovenia, Switzerland, United Kingdom, United States
Europe (low income), Russia & Central Asia	14 (13.1)	Afghanistan, Albania, Belarus, Bosnia and Herzegovina, Croatia, Republic of Georgia, Kazakhstan, Kyrgyzstan, Moldova, North Macedonia, Russia, Serbia, Ukraine, Uzbekistan
Latin America & the Caribbean	13 (12.1)	Colombia, Costa Rica, Cuba, Dominican Republic, El salvador, Guatemala, Haiti, Honduras, Jamaica, Mexico, Panama, Trinidad and Tobago, Venezuela
Mediterranean Basin	5 (4.7)	Cyprus, Greece, Italy, Portugal, Spain
Middle East & North Africa	21 (19.6)	Armenia, Azerbaijan, Bahrain, Egypt, Iran, Iraq, Israel, Jordan, Kuwait, Lebanon, Libya, Mali, Mauritania, Morocco, Oman, Qatar, Saudi Arabia, South Sudan, Tunisia, Turkey, United Arab Emirates
Nordic countries	4 (3.7)	Denmark, Finland, Norway, Sweden
South Asia	4 (3.7)	Bangladesh, India, Pakistan, Sri Lanka
Sub-Saharan Africa	13 (12.1)	Benin, Cameroon, Central African Republic, Cote d'Ivoire, Equatorial Guinea, Ethiopia, The Gambia, Ghana, Guinea, Nigeria, Senegal, Togo, Uganda
Total	107 (100.0)	

Table 2 summarized the inherent characteristics, namely, the number of tests for COVID-19 performed per one million population (COVID-19 test rate); demographic, socio-economic features; Global Health Security capabilities; healthcare capacities; and, personal health-related features, of the 107 countries. COVID-19 test rate was 55,710.0 (25–75th percentile 14,451.0–136,931.0). The proportion of female was 50.4% (25–75th percentile 49.8–51.2). The median age was 32.5 years (25–75th percentile 25.6–41.7), and the proportion of the population over 65 years of age was 8.6% (25–75th percentile 4.2–17.6). The population density (P/km^2^) was 103.0 (25–75th percentile 56.0–219.0) and the proportion of urban population was 0.7 (25–75th percentile 0.5–0.8).

**Table 2:** Characteristics of total selected countries.

As of 14 September 2020 (N = 107)	Median	Minimum	25th percentile	75th percentile	Maximum
COVID-19 test rate	55,710.0	1073.0	14,451.0	136,931.0	819,779.0
**Demographic variables**					
Female (% of total population)	50.4	24.7	49.8	51.2	54.4
Land area (km^2^)	176,520.0	700.0	51,060.0	510,890.0	16,376,870.0
Median age (years of age)	32.5	16.3	25.6	41.7	48.4
Over 65 years of age (% of total population)	8.6	1.2	4.2	17.6	28.0
Population (N)	11,193,725.0	1,207,359.0	5,421,241.0	40,222,493.0	1,439,323,776.0
Population density (P/km^2^)	103.0	2.0	56.0	219.0	8358.0
Urban population (of total population)	0.7	0.2	0.5	0.8	1.0
**Socio-economic variables**					
Education index	0.7	0.3	0.6	0.8	0.9
GDP per capita (current US$)	7808.2	467.9	2574.9	23,504.0	81,993.7
Gini index	34.7	24.2	31.5	40.8	56.2
International tourism, receipts (% of total exports)	7.4	0.2	3.9	16.1	53.4
Unemployment (% of total labor force)	5.3	0.1	3.4	8.9	21.6
**Global Health Security capabilities**					
Overall GHSI	44.2	16.2	35.5	55.4	83.5
GHSI1: Prevention	40.9	1.9	28.1	52.9	83.1
GHSI2: Early Detection and Reporting	48.5	4.4	39.5	71.2	98.2
GHSI3: Rapid Response	44.0	17.6	32.6	52.0	91.9
GHSI4: Health System	31.6	5.0	19.5	45.7	73.8
GHSI5: Compliance	52.8	25.8	44.2	61.1	85.3
GHSI6: Risk Environment	57.0	22.1	46.8	69.6	87.1
**Healthcare capacity**					
Healthcare Access and Quality Index	72.0	28.6	55.3	81.7	91.8
Health expenditure (% of GDP)	6.4	1.2	4.4	8.2	17.1
Hospital beds (per 1000 people)	2.7	0.1	1.2	4.7	13.4
Nurses (per 1000 people)	4.2	0.1	1.4	7.3	19.5
Out-of-pocket expenditure (% of current health expenditure)	33.5	6.7	19.1	49.6	84.3
Physicians (per 1000 people)	2.3	0.1	0.7	3.3	8.4
**Personal health-related variables**					
Alcohol consumption (%)	7.1	0.0	2.7	10.5	15.2
Diabetes prevalence (%)	6.8	1.0	5.4	9.2	19.9
Obesity prevalence (%)	21.5	2.1	10.2	25.4	37.9
Smoking prevalence (%)	23.6	3.7	14.2	28.2	45.5
WASH: Water	97.1	40.7	89.3	99.0	99.0
WASH: Sanitation	94.2	7.3	75.8	99.0	99.0

*COVID-19 test rate* number of COVID-19 tests performed per one million population, *GDP* Gross Domestic Product, *GHSI* Global health Security Index, *WASH: Water* index that assesses the safety and accessibility to water, *WASH: Sanitation* index that assesses the facility sanitation.

The median education index was 0.7 (25–75th percentile 0.6–0.8). The GDP per capita was US$ 7808.2 (25–75th percentile 2574.9–23,504.0), and Gini index was 34.7 (25–75th percentile 31.5–40.8). The proportions of international tourism receipts and unemployment was 7.4% (25–75th percentile 3.9–16.1) and 5.3% (25–75th percentile 3.4–8.9), respectively. The overall GHSI was 44.2 (25–75th percentile 35.5–55.4). The GHSI assessing the risk environment (GHSI6) had the highest score (57.0, 25–75th percentile 46.8‒69.6) among the six categories whereas the index assessing health system (GHSI4) had the lowest score (31.6, 25–75th percentile 19.5–45.7).

The HAQ score was 72.0 (25–75th percentile 55.3–81.7). The percentage of health expenditure within the GDP was 6.4% (25–75th percentile 4.4–8.2), and the percentage of out-of-pocket expenditure was 33.5% (25–75th percentile 19.1–49.6). The number of hospital beds, nurses, and physicians per 1000 people were 2.7 (25–75th percentile 1.2–4.7), 4.2 (25–75th percentile 1.4–7.3), and 2.3 (25–75th percentile 0.7–3.3), respectively. The prevalence of alcohol consumption and smoking were 7.1% (25–75th percentile 2.7–10.5) and 23.6% (25–75th percentile 14.2–28.2). The prevalence of diabetes and obesity were 6.8% (25–75th percentile 5.4–9.2) and 21.5% (25–75th percentile 10.2–25.4). The WASH index for water safety was 97.1 (25–75th percentile 89.3–99.0) and index for facility sanitation was 94.2 (25–75th percentile 75.8–99.0).

### COVID-19 incidence and case-fatality ratio as of 14 September 2020

The COVID-19 health-related outcomes among 107 countries as of 14 September 2020 are summarized in Table 3. The median value for incidence was 2583.0 (25–75th percentile 783.0–6261.0) and the maximum was 43,358.0, while the minimum was 3.0. The median value of deaths per one million population was 55.0 (25–75th percentile 11.0–152.0) and the maximum value was 855.0 whereas three countries, Laos, Mongolia, and Cambodia, had no deaths. The median CFR was 2.1% (25–75th percentile 1.3–3.2) and the maximum was 12.4% whereas the minimum was 0.0%.

**Table 3:** COVID-19 health-related outcomes of total selected countries.

As of 14 September 2020 (N = 107)	Median	Mean	Minimum	25th percentile	75th percentile	Maximum
COVID-19 incidence	2583.0	4858.0	3.0	783.0	6261.0	43,358.0
COVID-19 mortality	55.0	123.2	0.0	11.0	152.0	855.0
COVID-19 case-fatality ratio (%)	2.1	2.7	0.0	1.3	3.2	12.4

*COVID-19 incidence* total confirmed cases of COVID-19 per one million population, *COVID-19 mortality* deaths due to COVID-19 per one million population.

**Figure 2:**
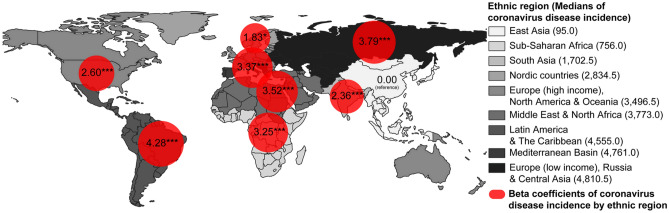
Medians and beta coefficients of COVID-19 incidence by ethnic region in 107 northern hemisphere countries. The size of red circle indicates the beta coefficients, which were determined by using multiple linear regression analysis on log transformed COVID-19 incidence, having “East Asia” region as a reference (*p < 0.05, **p < 0.01, ***p < 0.001).

**Figure 3:**
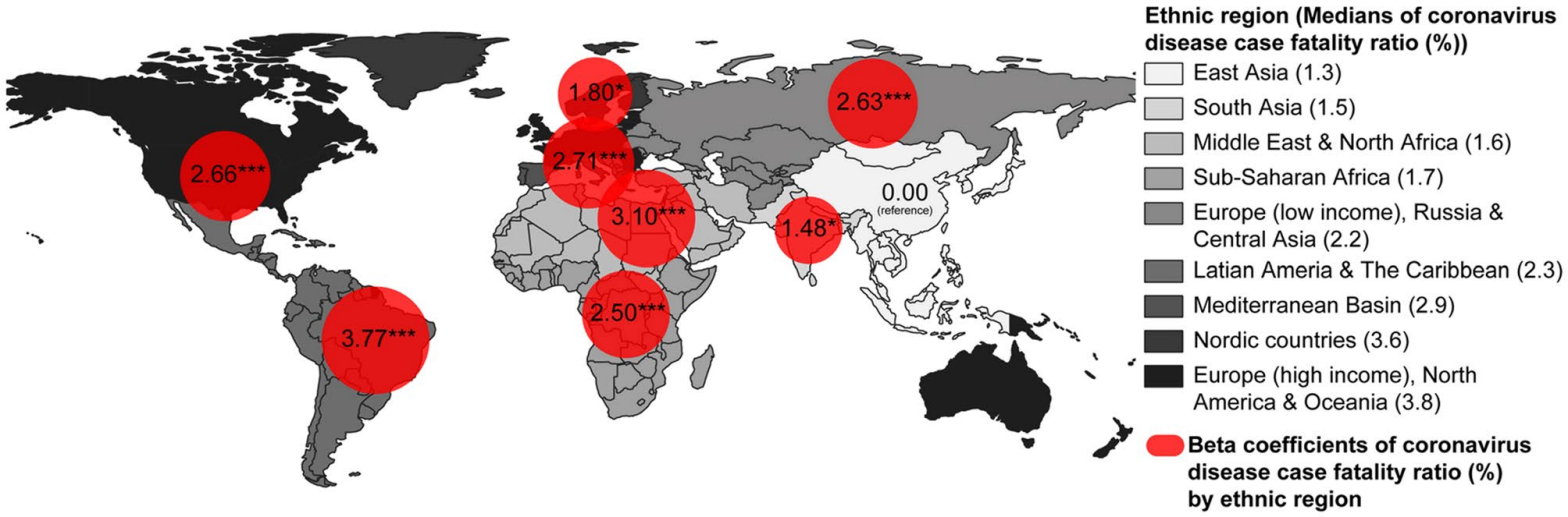
Medians and beta coefficients of COVID-19 case-fatality ratio (%) by ethnic region in 107 northern hemisphere countries. The size of red circle indicates the beta coefficients, which were determined by using multiple linear regression analysis on log transformed COVID-19 case-fatality ratio, having “East Asia” region as a reference (*p < 0.05, **p < 0.01, ***p < 0.001).

The median values of incidence and CFR across ethnic region are summarized in Figs. 2 and 3 and Table S1 in Supplement. The median value of incidence in “East Asia” was 95.0 (25–75th percentile 33.0–1223.5) whereas that in “Europe (low income), Russia and Central Asia” was 4810.5 (25–75th percentile 2828.0–7356.5). The CFR in “East Asia” was 1.3 (25–75th percentile 0.0–1.8) whereas that in “Europe (high income), North America and Oceania” was 3.8 (25–75th percentile 2.4–6.5).

### Factors related to COVID-19 incidence

The results of the multiple linear regression analysis to investigate the significant factors affecting COVID-19 incidence are presented in Table 4. The explanatory power of the model was 63.7% (adjusted ${R}^{2}$ = 0.637). Ethnic region (*p* < 0.05), GHSI4 (β 0.50, 95% CI 0.14–0.87), population density (β 0.35, 95% CI 0.10–0.60), and WASH index for water safety (β 0.51, 95% CI 0.19–0.84) had positive associations with incidence, even after adjusting for the national demographic and economic features and COVID-19 test rates. Specifically, all other ethnic regions had significantly higher incidences than “East Asia.” “Latin America and the Caribbean” region had the highest beta coefficient among the regions (β 4.28, 95% CI 3.32–5.23). Ethnic region had the highest partial R-squared statistics among the factors (partial ${R}^{2}$ = 0.545). If the country had a higher GHSI4, population density, and WASH index for water safety, it was likely to have a higher incidence and ethnic region explained the largest part of the model.

**Table 4:** Multiple linear regression analysis on log transformed COVID-19 incidence.

As of 14 September 2020 (N = 107)	β	SE	(95% CI)	*p* Value	Partial $${{\varvec{R}}}^{2}$$
COVID-19 test rate	0.09	0.15	(− 0.21‒0.39)	0.561	0.004
Ethnic region					0.545
East Asia	ref				
Europe (high income), North America & Oceania	2.60	0.50	(1.61‒3.59)	< 0.001	
Europe (low income), Russia & Central Asia	3.79	0.47	(2.87‒4.70)	< 0.001	
Latin America & the Caribbean	4.28	0.49	(3.32‒5.23)	< 0.001	
Mediterranean Basin	3.37	0.66	(2.07‒4.67)	< 0.001	
Middle East & North Africa	3.52	0.45	(2.63‒4.41)	< 0.001	
Nordic countries	1.83	0.77	(0.31‒3.35)	0.020	
South Asia	2.36	0.62	(1.13‒3.58)	< 0.001	
Sub-Saharan Africa	3.25	0.54	(2.20‒4.31)	< 0.001	
Female (% of total population)	− 0.31	0.16	(− 0.62‒0.00)	0.052	0.042
GDP per capita (current US$)	0.11	0.26	(− 0.40‒0.62)	0.669	0.002
GHSI4: Health System	0.50	0.19	(0.14‒0.87)	0.008	0.077
Median age (years of age)	− 0.04	0.27	(− 0.57‒0.49)	0.881	< 0.001
Nurses (per 1000 people)	0.44	0.24	(− 0.04‒0.92)	0.075	0.036
Out-of-pocket expenditure (% of current health expenditure)	0.27	0.15	(− 0.03‒0.56)	0.077	0.035
Physicians (per 1000 people)	− 0.26	0.18	(− 0.62‒0.09)	0.152	0.023
Population density (P/km^2^)	0.35	0.13	(0.10‒0.60)	0.008	0.078
WASH: Water	0.51	0.17	(0.19‒0.84)	0.003	0.097
Adjusted $${R}^{2}$$	0.637				

*COVID-19 incidence* total confirmed cases of COVID-19 per one million population, *β* beta coefficients, *SE* standard error, *95% CI* 95% confidence interval, *COVID-19 test rate* number of COVID-19 tests performed per one million population, *GDP* Gross Domestic Product, *GHSI* Global Health Security Index, *WASH: Water*, index that assesses the safety and accessibility to water.

### Factors related to COVID-19 case-fatality ratio

The results of the multiple linear regression analysis to investigate the significant factors influencing CFR are presented in Table 5. The model had 49.9% of explanatory power (adjusted ${R}^{2}$ = 0.499). The factors that had positive associations with the CFR were ethnic region (*p* < 0.05), GHSI4 (β 0.53, 95% CI 0.14–0.92), the proportion of the population over 65 years of age (β 0.71, 95% CI 0.19–1.24) whereas the number of physicians (β − 0.37, 95% CI − 0.69 to − 0.06) and the number of international tourism receipts (β − 0.23, 95% CI − 0.43 to − 0.03) had a negative association with the CFR. Specifically, the CFRs of all the other ethnic regions were significantly higher than that of “East Asia,” even after adjusting for sex, age, economic status, and COVID-19 incidence. The beta coefficient of “Latin America and the Caribbean” region was the highest among the ethnic regions (β 3.77, 95% CI 2.62–4.92). Ethnic region had the highest partial R-squared statistics among the factors (partial ${R}^{2}$ = 0.372). Countries with higher GHSI4, higher proportions of population over 65 years of age, fewer international tourism receipts, and fewer physicians were likely to have higher CFRs and ethnic region explained the largest part of the model.

**Table 5:** Multiple linear regression analysis on log transformed COVID-19 case-fatality ratio.

As of 14 September 2020 (N = 107)	β	SE	(95% CI)	*p* Value	Partial $${{\varvec{R}}}^{2}$$
Alcohol consumption (%)	− 0.29	0.17	(− 0.63‒0.05)	0.098	0.033
COVID-19 incidence	− 0.16	0.15	(− 0.46‒0.14)	0.290	0.013
Education index	− 0.16	0.25	(− 0.65‒0.32)	0.512	0.005
Ethnic region					0.372
East Asia	ref				
Europe (high income), North America & Oceania	2.66	0.60	(1.49‒3.83)	< 0.001	
Europe (low income), Russia & Central Asia	2.63	0.52	(1.61‒3.64)	< 0.001	
Latin America & the Caribbean	3.77	0.59	(2.62‒4.92)	< 0.001	
Mediterranean Basin	2.71	0.71	(1.31‒4.11)	< 0.001	
Middle East & North Africa	3.10	0.64	(1.84‒4.36)	< 0.001	
Nordic countries	1.80	0.77	(0.29‒3.30)	0.022	
South Asia	1.48	0.58	(0.33‒2.62)	0.013	
Sub-Saharan Africa	2.50	0.46	(1.60‒3.39)	< 0.001	
Female (% of total population)	− 0.08	0.16	(− 0.41‒0.24)	0.610	0.003
GDP per capita (current US$)	− 0.08	0.19	(− 0.46‒0.29)	0.659	0.002
GHSI1: Prevention	0.37	0.22	(− 0.06‒0.81)	0.095	0.033
GHSI4: Health System	0.53	0.20	(0.14‒0.92)	0.010	0.078
GHSI5: Compliance	0.05	0.14	(− 0.22‒0.32)	0.708	0.002
International tourism, receipts (% of total exports)	− 0.23	0.10	(− 0.43 to − 0.03)	0.028	0.057
Obesity prevalence (%)	− 0.39	0.22	(− 0.83‒0.05)	0.085	0.035
Out-of-pocket expenditure (% of current health expenditure)	0.23	0.14	(− 0.05‒0.51)	0.110	0.031
Over 65 years of age (%)	0.71	0.27	(0.19‒1.24)	0.009	0.079
Physicians (per 1000 people)	− 0.37	0.16	(− 0.69 to − 0.06)	0.024	0.060
Population density (P/km^2^)	− 0.21	0.11	(− 0.43‒0.00)	0.058	0.043
Unemployment (% of total labor force)	0.11	0.13	(− 0.14‒0.37)	0.397	0.009
Adjusted $${R}^{2}$$	0.499				

*β* beta coefficients, *SE* standard error, *95% CI* 95% confidence interval, *COVID-19 incidence* total confirmed cases of COVID-19 per one million population, *GDP* Gross Domestic Product, *GHSI* Global Health Security Index.

## Discussion

An analysis was conducted with publicly available data to identify the factors associated with COVID-19 incidence and CFR. Possible factors, namely, COVID-19 test rate; geographical, demographical, and socio-economic variables; degree of preparedness for epidemics; healthcare capacity; and, personal health-related variables, were evaluated.

Ethnic region was the most influential factor for both COVID-19 incidence and CFR, even after adjusting for the national demographic and economic features and COVID-19 test rates/incidence. The results of the sub-analysis including countries in both hemispheres also showed that ethnic region accounts for the largest part in the incidence (partial ${R}^{2}$ = 0.511) and CFR models (partial ${R}^{2}$ = 0.322) (Tables S4 and S5 in Supplement). Furthermore, sub-analyses by each ethnic region did not reveal any significant factors related to incidence and CFR consistently (Tables S6 and S7 in Supplement). Our results are possible to support the hypothesis that East Asia could have evolved for a long time to be more resistant to SARS-CoV-2, suggested by Yamamoto and Bauer^2^. Yamamoto and Bauer^2^ proposed that, differences in (1) socio-behavioral aspects, (2) virulency of viruses, (3) evolutionary history related to selection of people by the virus, or (4) hygienic conditions could cause discrepancies in COVID-19 outcomes between Central Europe and East Asia. In our results, ethnic region was the most influential features explaining the international variation of the disease, even after considering socio-behavioral aspects and hygienic aspects, with the WASH index, as possible factors. As COVID-19 control policies were implemented to constrain socio-behavioral aspects, national differences in policies could partly explain the differences in incidence^2,19^. However, the national differences in policies could not fully explain the differences in the CFRs across countries^2^. Chaudhry et al.^19^ also suggested that government actions, such as rapid border closing and complete lockdowns, could not sufficiently explain COVID-19 mortality. Furthermore, since there are insufficient virological studies investigating SARS-CoV-2 worldwide^2^, the hypothesis that highlighted the differences in pathogenicity of viruses across regions is hardly supported. Therefore, our findings could support the ‘evolutionary hypothesis’ among the four hypotheses to explain these regional variations suggested by Yamamoto and Bauer^2^. That is, the difference in native susceptibility of the hosts in each region may be a possible factor to explain these regional variations of incidence and fatality of COVID-19. Asians living in ‘Asian ethnic region’ including Chinese may have lower susceptibility to SARS-CoV-2, for any reason including the possibility of exposure to a pathogen with a similar antigenicity in the past. However, our data and analysis in this study may be insufficient to rule out other possible hypotheses and explanations. We are not against the results of previous studies^34^ that the impact of the effective control measures against COVID-19 in East Asia could have resulted in lower incidence and CFR. As our study being country-level ecological study, we aim to suggest a hypothesis, not to prove hypothesis. Therefore, further studies at the individual levels are required to derive direct evidence for different susceptibilities to COVID-19 across ethnic regions, considering collinearity between ethnic region and control measures.

GHSI4, which evaluated the health system, was associated with a higher COVID-19 incidence and CFR. Our results support the argument that GHSI is not sufficiently predictive of pandemic response^35,36^, and additional factors that better estimate pandemic preparedness should be embedded in the index^36^. However, we should be cautious while interpreting the predictiveness of GHSI for the vulnerability to the epidemic as the COVID-19 pandemic is still ongoing.

Countries with better water safety levels were likely to have higher incidence. These results support the hypothesis that poorer hygienic conditions are associated with higher resistance to infectious disease^2^. However, the observed negative effects of the WASH Index should be interpreted with caution. The association between water security and incidence might have resulted because countries with high water security usually had high economic statuses, given that GDP per capita and WASH index for water safety had a positive correlation (r = 0.47, *p* < 0.001). Therefore, the authors are not convinced of the negative effect of water safety and support that water security should be ensured for tackling the pandemic^37^.

Countries with higher population densities were expected to have higher incidences. In common perception, dense areas could be vulnerable to closer contact, which leads to higher caseloads in directly transmitted infectious diseases. Our study supports this common perception, which is also supported by Bhadra et al.^20^ and Coşkun et al.^21^. However, a study that analyzed 913 U.S. metropolitan counties^22^ disputed this perception by showing that the connectivity between counties was significantly associated with incidence rather than the population density. As studies are usually performed within countries^20–22^, further studies at the country level are needed to clarify whether population density is associated with the disease outcomes.

As examined by several other studies^19,38^, older age was associated with a higher CFR. Older patients with COVID-19 are more vulnerable to progress to severe disease^39^ and a greater number of patients with severe disease could burden the national economy and healthcare capacity. Therefore, the government should have great interest in older patients with COVID-19.

Countries with fewer healthcare professionals, especially physicians, were vulnerable to CFR. It is possible to consider that an increase in CFR, resulting from the lack of healthcare professionals, could lead to the collapse of the healthcare system. Retaining a sufficient number of healthcare workers is essential to win this war^40^. Therefore, the government should secure the safety and well-being of healthcare professionals in physical and psychological aspects^40,41^.

Countries with higher usual tourism receipts were likely to have lower CFRs. Contrastingly, Farzanegan et al.^23^ suggested that countries with higher inbound and outbound tourism are more likely to have higher number of confirmed cases and deaths. Most European countries enforced border control measures at a later stage as compared to Asia-Pacific countries^42^. Since the extraction date of COVID-19 outbreak data we used is about five months later than that of a previous study^23^, it is possible for the effect of border control to be fully reflected in our study. However, effect of border control could not be fully considered, further studies which consider the characteristics of border controls implemented by countries are required.

Our study has several limitations. As COVID-19 pandemic is still ongoing, the data we used has limitation with respect to reflecting the current situation. Because the information related to COVID-19 was extracted only once, i.e., on 14 September 2020, information after this date cannot be applied in our analysis. However, by setting 14 September 2020 as data capture date, we could consider that most of the countries had gone through the first wave of COVID-19^17,18^ and we could reduce the chances of biased results because of possible cocirculation of flu and COVID-19^18^, and because of possible effect of vaccination. We did not include national control measures as potential factors, as mitigation policies themselves have limitations in comparing effectiveness. Specifically, each country had various kinds of policies at different intensities^43,44^, different initiation times^43,44^, and various degrees of compliance of the public to the policy^45–47^. Age-standardization, which is useful to fairly compare the disease outcomes across countries^48^, could not be implemented in our study. This was because each country reported the outcomes with different age standards, and some countries did not report based on age group. However, including age-representing variables in the analysis models must have adjusted the differences in age structure among countries to some degree. Finally, we hardly support a definitive judgement on the effect of ethnicity across countries, as the categories of ethnic region we used were not based on social consent but were ones used by a single published article^24^. However, because social standards in ethnic category are absent, the ethnic grouping we used was the best option to handle the ethnic categories. Genetic factors could not be investigated in our study because data regarding genetic factors related to COVID-19 was unavailable.

This study is meaningful in examining the association of ethnicity with COVID-19 health-related outcomes at the country level and highlighting that ethnicity could largely explain COVID-19 incidence and CFR. Moreover, the authors consider that this work could be used as a trigger for further research investigating the effect of different genetic predispositions across ethnicities on COVID-19 outcomes.

## Supplementary Information

Supplementary Tables.

## Data Availability

Information on COVID-19 health-related outcomes is open to public. Data download is available in the following website: https://www.worldometers.info/coronavirus. This research has been conducted using COVID-19 health-related outcomes on 14 September 2020. Information on country-level indices, including demographic, and socio-economic features, global health security index, healthcare capacity, and health behaviors, is publicly available. Data could be downloaded from following websites: World Bank Open data (https://data.worldbank.org); Human Development Data (1990–2018) (http://hdr.undp.org/en/data); Countries in the world by population (https://www.worldometers.info/world-population/population-by-country); 2019 Global Health Security Index (https://www.ghsindex.org); Global Burden of Disease Study 2015 (GBD 2015) (http://ghdx.healthdata.org/record/ihme-data/gbd-2015-healthcare-access-and-quality-index-1990-2015); World Health Data Platform (https://www.who.int/data); and Water, sanitation & hygiene (WASH) data (https://data.unicef.org/resources/dataset/drinking-water-sanitation-hygiene-database). Data used in this work are available upon request to the corresponding author. The Shapefile used for Figs. 2 and 3 was obtained from “Admin 0-Countries” of Natural Earth (https://www.naturalearthdata.com/downloads/110m-cultural-vectors/). The data to create maps for academic publishing are freely available (Term of use: https://www.naturalearthdata.com/about/terms-of-use/).
