# Supplementary material for: Factors associated with the difference between the incidence and case-fatality ratio of coronavirus disease 2019 by country
Source: Sci Rep. 2021 Sep 23;11:18938. doi: 10.1038/s41598-021-98378-x (PMC8460795; doi:10.1038/s41598-021-98378-x)
Supplement: Supplementary file 1 — Supplementary Tables. [file 41598_2021_98378_MOESM1_ESM.docx]

**Supplementary Information**

**Title**

Factors Associated with the Difference Between the Incidence and Case-Fatality Ratio of Coronavirus Disease 2019 by Country

**Authors**

Jeehyun Kim^1,2,3^, Kwan Hong^1,2^, Sujin Yum^1,2^, Raquel Elizabeth Gómez Gómez^1,2^, Jieun Jang^1^, Sun Hee Park^4^, Young June Choe^5^, Sukhyun Ryu^6^, Dae Won Park^7^, Young Seok Lee^8^, Heeyoung Lee^9^, Dong Hyun Kim^10^, Dong-Hyun Kim^11^, and Byung Chul Chun^1,2,3,*^

^1^Department of Preventive Medicine, Korea University College of Medicine, Seoul, Republic of Korea

^2^Graduate School of Public Health, Korea University, Seoul, Republic of Korea

^3^Transdisciplinary Major in Learning Health Systems, Department of Healthcare Sciences, Graduate School, Korea University, Seoul, Republic of Korea

^4^Division of Infectious Diseases, Department of Internal Medicine, College of Medicine, The Catholic University of Korea, Seoul, Republic of Korea

^5^Department of Pediatrics, Korea University Anam Hospital, Seoul, Republic of Korea

^6^Department of Preventive Medicine, Konyang University College of Medicine, Daejeon, Republic of Korea

^7^Division of Infectious Diseases, Department of Internal Medicine, Korea University Ansan Hospital, Ansan, Republic of Korea

^8^Division of Pulmonary, Allergy, and Critical Care Medicine, Department of Internal Medicine, Korea University Guro Hospital, Seoul, Republic of Korea

^9^Center for Preventive Medicine and Public Health, Seoul National University Bundang Hospital, Seongnam, Republic of Korea

^10^Department of Pediatrics, Inha University School of Medicine, Incheon, Republic of Korea

^11^Department of Social and Preventive Medicine, Hallym University College of Medicine, Chuncheon, Gangwon, Republic of Korea

^*^chun@korea.ac.kr

**Supplementary Table S1.** COVID-19 health-related outcomes by ethnic region as of 14 September 2020 (N=107). *COVID-19 Incidence* total confirmed cases of COVID-19 per one million population, *COVID-19 mortality* deaths due to COVID-19 per one million population, *Min* minimum, *Max* maximum, *25th* 25th percentile, *75th* 75th percentile.

| **Ethnic region** | **COVID-19 incidence** | | | | | | **COVID-19 mortality** | | | | | | **COVID-19 case-fatality ratio (%)** | | | | | |
| --- | --- | --- | --- | --- | --- | --- | --- | --- | --- | --- | --- | --- | --- | --- | --- | --- | --- | --- |
|  | **Median** | **Mean** | **Min** | **25th** | **75th** | **Max** | **Median** | **Mean** | **Min** | **25th** | **75th** | **Max** | **Median** | **Mean** | **Min** | **25th** | **75th** | **Max** |
| East Asia | 95.0 | 1,203.6 | 3.0 | 33.0 | 1,223.5 | 9,797.0 | 3.0 | 6.4 | 0.0 | 0.2 | 9.0 | 40.0 | 1.3 | 1.4 | 0.0 | 0.0 | 1.8 | 5.4 |
| Europe (high income), North America & Oceania | 3,496.5 | 4,388.9 | 783.0 | 1,825.8 | 5,438.0 | 20,239.0 | 108.0 | 229.8 | 7.0 | 50.5 | 363.8 | 855.0 | 3.8 | 4.6 | 0.7 | 2.4 | 6.5 | 11.3 |
| Europe (low income), Russia & Central Asia | 4,810.5 | 5,036.6 | 558.0 | 2,828.0 | 7,356.5 | 10,659.0 | 85.5 | 117.0 | 5.0 | 50.3 | 174.5 | 311.0 | 2.2 | 2.2 | 0.8 | 1.4 | 2.9 | 4.1 |
| Latin America & the Caribbean | 4,555.0 | 6,558.2 | 414.0 | 1,685.0 | 10,201.5 | 23,508.0 | 121.0 | 183.4 | 10.0 | 18.0 | 329.0 | 546.0 | 2.3 | 2.9 | 0.8 | 1.4 | 3.1 | 10.6 |
| Mediterranean Basin | 4,761.0 | 5,181.6 | 1,262.0 | 1,267.0 | 9,306.5 | 12,334.0 | 183.0 | 291.0 | 18.0 | 23.5 | 612.5 | 636.0 | 2.9 | 4.8 | 1.4 | 1.9 | 8.8 | 12.4 |
| Middle East & North Africa | 3,773.0 | 9,528.9 | 144.0 | 1,270.0 | 16,194.5 | 43,358.0 | 55.0 | 91.7 | 2.0 | 34.5 | 127.5 | 309.0 | 1.6 | 1.9 | 0.2 | 0.7 | 2.3 | 5.8 |
| Nordic countries | 2,834.5 | 3,943.0 | 1,548.0 | 1,720.5 | 7,274.0 | 8,555.0 | 85.0 | 199.3 | 49.0 | 52.0 | 460.8 | 578.0 | 3.6 | 4.0 | 2.2 | 2.4 | 6.1 | 6.8 |
| South Asia | 1,702.5 | 1,765.0 | 151.0 | 453.3 | 3,139.3 | 3,504.0 | 29.0 | 29.2 | 0.6 | 7.7 | 50.8 | 58.0 | 1.5 | 1.4 | 0.4 | 0.6 | 2.0 | 2.1 |
| Sub-Saharan Africa | 756.0 | 905.2 | 104.0 | 230.5 | 1,190.5 | 3,539.0 | 9.0 | 14.5 | 1.0 | 4.5 | 17.0 | 59.0 | 1.7 | 1.6 | 0.6 | 0.9 | 2.1 | 3.0 |

**Supplementary Table S2.** Univariate linear regression analysis on log transformed COVID-19 incidence of total selected countries. *COVID-19 incidence* total confirmed cases of COVID-19 per one million population, *β* beta coefficients, *SE* standard error, *95% CI* 95% confidence interval, *COVID-19 test rate* number of COVID-19 tests performed per one million population, *GDP* Gross Domestic Product, *GHSI* Global Health Security Index, *WASH: Water* index that assesses the safety and accessibility to water, *WASH: Sanitation* index that assesses the facility sanitation.

| **As of 14 September 2020 (N=107)** | **β** | **SE** | **(95% CI)** | **p-value** |
| --- | --- | --- | --- | --- |
| COVID-19 test rate | 0.75 | 0.15 | (0.45‒1.04) | <0.001 |
| **Ethnic region** |  |  |  |  |
| East Asia | ref. |  |  |  |
| Europe (high income), North America & Oceania | 3.04 | 0.48 | (2.09‒3.99) | <0.001 |
| Europe (low income), Russia & Central Asia | 3.23 | 0.52 | (2.20‒4.25) | <0.001 |
| Latin America & the Caribbean | 3.22 | 0.53 | (2.17‒4.26) | <0.001 |
| Mediterranean Basin | 3.15 | 0.71 | (1.75‒4.55) | <0.001 |
| Middle East & North Africa | 3.20 | 0.48 | (2.26‒4.14) | <0.001 |
| Nordic countries | 3.03 | 0.78 | (1.51‒4.55) | <0.001 |
| South Asia | 1.97 | 0.78 | (0.45‒3.49) | 0.013 |
| Sub-Saharan Africa | 1.36 | 0.53 | (0.32‒2.41) | 0.012 |
| **Demographic variables** |  |  |  |  |
| Female (% of total population) | -0.48 | 0.16 | (-0.80‒-0.17) | 0.003 |
| Land area (km²) | 0.06 | 0.17 | (-0.27‒0.39) | 0.734 |
| Median age (years of age) | 0.53 | 0.16 | (0.21‒0.84) | 0.001 |
| Over 65 years of age (% of total population) | 0.36 | 0.16 | (0.04‒0.68) | 0.031 |
| Population (N) | -0.22 | 0.17 | (-0.54‒0.11) | 0.195 |
| Population density (P/km²) | 0.22 | 0.17 | (-0.10‒0.55) | 0.184 |
| Urban population (of total population) | 0.92 | 0.14 | (0.64‒1.19) | <0.001 |
| **Socio-economic variables** |  |  |  |  |
| Education index | 0.68 | 0.15 | (0.38‒0.99) | <0.001 |
| GDP per capita (current US$) | 0.68 | 0.15 | (0.38‒0.98) | <0.001 |
| Gini index | -0.09 | 0.17 | (-0.42‒0.23) | 0.575 |
| International tourism, receipts (% of total exports) | -0.12 | 0.17 | (-0.45‒0.21) | 0.471 |
| Unemployment (% of total labor force) | 0.30 | 0.17 | (-0.03‒0.62) | 0.077 |
| **Global Health Security Capabilities** |  |  |  |  |
| Overall GHSI | 0.28 | 0.17 | (-0.04‒0.61) | 0.093 |
| GHSI1: Prevention | 0.37 | 0.16 | (0.05‒0.69) | 0.025 |
| GHSI2: Early Detection and Reporting | 0.04 | 0.17 | (-0.29‒0.37) | 0.807 |
| GHSI3: Rapid Response | 0.18 | 0.17 | (-0.15‒0.51) | 0.280 |
| GHSI4: Health System | 0.45 | 0.16 | (0.14‒0.77) | 0.006 |
| GHSI5: Compliance | -0.17 | 0.17 | (-0.50‒0.15) | 0.298 |
| GHSI6: Risk Environment | 0.62 | 0.16 | (0.31‒0.92) | <0.001 |
| **Healthcare capacity** |  |  |  |  |
| Healthcare Access and Quality Index | 0.76 | 0.15 | (0.47‒1.06) | <0.001 |
| Health expenditure (% of GDP) | 0.44 | 0.16 | (0.12‒0.76) | 0.008 |
| Hospital beds (per 1,000 people) | 0.21 | 0.17 | (-0.11‒0.54) | 0.206 |
| Nurses (per 1,000 people) | 0.61 | 0.16 | (0.30‒0.92) | <0.001 |
| Out-of-pocket expenditure (% of current health expenditure) | -0.47 | 0.16 | (-0.78‒-0.15) | 0.005 |
| Physicians (per 1,000 people) | 0.56 | 0.16 | (0.25‒0.87) | 0.001 |
| **Personal health-related variables** |  |  |  |  |
| Alcohol consumption (%) | 0.00 | 0.17 | (-0.33‒0.33) | 0.993 |
| Diabetes prevalence (%) | 0.49 | 0.16 | (0.17‒0.80) | 0.003 |
| Obesity prevalence (%) | 1.02 | 0.13 | (0.76‒1.29) | <0.001 |
| Smoking prevalence (%) | -0.04 | 0.17 | (-0.37‒0.29) | 0.810 |
| WASH: Water | 0.75 | 0.15 | (0.46‒1.05) | <0.001 |
| WASH: Sanitation | 0.76 | 0.15 | (0.46‒1.05) | <0.001 |

**Supplementary Table S3.** Univariate linear regression analysis on log transformed COVID-19 case-fatality ratio of total selected countries. *β* beta coefficients, *SE* standard error, *95% CI* 95% confidence interval, *COVID-19 incidence* total confirmed cases of COVID-19 per one million population*, GDP* Gross Domestic Product, *GHSI* Global Health Security Index, *WASH: Water* index that assesses the safety and accessibility to water, *WASH: Sanitation* index that assesses the facility sanitation.

| **As of 14 September 2020 (N=107)** | **β** | **SE** | **(95% CI)** | **p-value** |
| --- | --- | --- | --- | --- |
| COVID-19 incidence | -0.10 | 0.13 | (-0.35‒0.15) | 0.457 |
| **Ethnic region** |  |  |  |  |
| East Asia | ref. |  |  |  |
| Europe (high income), North America & Oceania | 2.44 | 0.40 | (1.65‒3.23) | <0.001 |
| Europe (low income), Russia & Central Asia | 1.78 | 0.44 | (0.93‒2.64) | <0.001 |
| Latin America & the Caribbean | 1.94 | 0.44 | (1.07‒2.81) | <0.001 |
| Mediterranean Basin | 2.40 | 0.60 | (1.23‒3.57) | <0.001 |
| Middle East & North Africa | 1.42 | 0.40 | (0.63‒2.20) | 0.001 |
| Nordic countries | 2.42 | 0.65 | (1.15‒3.69) | <0.001 |
| South Asia | 1.26 | 0.65 | (0.00‒2.53) | 0.054 |
| Sub-Saharan Africa | 1.47 | 0.44 | (0.60‒2.34) | 0.001 |
| **Demographic variables** |  |  |  |  |
| Female (% of total population) | 0.31 | 0.12 | (0.07‒0.56) | 0.013 |
| Land area (km²) | 0.13 | 0.13 | (-0.12‒0.38) | 0.318 |
| Median age (years of age) | 0.33 | 0.12 | (0.09‒0.57) | 0.009 |
| Over 65 years of age (% of total population) | 0.50 | 0.12 | (0.27‒0.73) | <0.001 |
| Population (N) | 0.14 | 0.13 | (-0.11‒0.39) | 0.283 |
| Population density (P/km²) | -0.36 | 0.12 | (-0.60‒-0.12) | 0.004 |
| Urban population (of total population) | 0.23 | 0.13 | (-0.01‒0.48) | 0.065 |
| **Socio-economic variables** |  |  |  |  |
| Education index | 0.28 | 0.12 | (0.03‒0.52) | 0.028 |
| GDP per capita (current US$) | 0.20 | 0.13 | (-0.05‒0.45) | 0.120 |
| Gini index | -0.09 | 0.13 | (-0.34‒0.16) | 0.473 |
| International tourism, receipts (% of total exports) | -0.16 | 0.13 | (-0.41‒0.09) | 0.218 |
| Unemployment (% of total labor force) | 0.33 | 0.12 | (0.08‒0.57) | 0.010 |
| **Global Health Security Capabilities** |  |  |  |  |
| Overall GHSI | 0.33 | 0.12 | (0.09‒0.57) | 0.009 |
| GHSI1: Prevention | 0.40 | 0.12 | (0.16‒0.63) | 0.002 |
| GHSI2: Early Detection and Reporting | 0.15 | 0.13 | (-0.09‒0.40) | 0.226 |
| GHSI3: Rapid Response | 0.21 | 0.13 | (-0.04‒0.45) | 0.106 |
| GHSI4: Health System | 0.41 | 0.12 | (0.17‒0.65) | 0.001 |
| GHSI5: Compliance | 0.32 | 0.12 | (0.08‒0.57) | 0.010 |
| GHSI6: Risk Environment | 0.25 | 0.13 | (0.00‒0.49) | 0.053 |
| **Healthcare capacity** |  |  |  |  |
| Healthcare Access and Quality Index | 0.33 | 0.12 | (0.08‒0.57) | 0.010 |
| Health expenditure (% of GDP) | 0.55 | 0.12 | (0.32‒0.77) | <0.001 |
| Hospital beds (per 1,000 people) | 0.16 | 0.13 | (-0.09‒0.41) | 0.221 |
| Nurses (per 1,000 people) | 0.34 | 0.12 | (0.10‒0.58) | 0.007 |
| Out-of-pocket expenditure (% of current health expenditure) | -0.21 | 0.13 | (-0.45‒0.04) | 0.107 |
| Physicians (per 1,000 people) | 0.24 | 0.13 | (-0.01‒0.49) | 0.058 |
| **Personal health-related variables** |  |  |  |  |
| Alcohol consumption (%) | 0.26 | 0.13 | (0.02‒0.51) | 0.040 |
| Diabetes prevalence (%) | -0.05 | 0.13 | (-0.30‒0.20) | 0.704 |
| Obesity prevalence (%) | 0.27 | 0.13 | (0.03‒0.52) | 0.032 |
| Smoking prevalence (%) | -0.06 | 0.13 | (-0.31‒0.19) | 0.660 |
| WASH: Water | 0.22 | 0.13 | (-0.03‒0.47) | 0.082 |
| WASH: Sanitation | 0.20 | 0.13 | (-0.05‒0.45) | 0.117 |

**Supplementary Table S4.** Multiple linear regression analysis on log transformed COVID-19 incidence for 136 countries, including countries in both northern and southern hemispheres. *COVID-19 incidence* total confirmed cases of COVID-19 per one million population, *β* beta coefficients, *SE* standard error, *95% CI* 95% confidence interval, *COVID-19 test rate* number of COVID-19 tests performed per one million population, *GDP* Gross Domestic Product, *GHSI* Global Health Security Index, *WASH: Water* index that assesses the safety and accessibility to water, *WASH: Sanitation* index that assesses the facility sanitation.

| **As of 14 September 2020 (N=136)** | **β** | **SE** | **(95% CI)** | **p-value** | **Partial** $\boldsymbol{R}^{\boldsymbol{2}}$ |
| --- | --- | --- | --- | --- | --- |
| COVID-19 test rate | 0.14 | 0.14 | (-0.14–0.41) | 0.327 | 0.008 |
| Ethnic region |  |  |  |  | 0.511 |
| East Asia | ref. |  |  |  |  |
| Europe (high income), North America & Oceania | 2.38 | 0.49 | (1.41–3.34) | <0.001 |  |
| Europe (low income), Russia & Central Asia | 3.48 | 0.47 | (2.56–4.39) | <0.001 |  |
| Latin America & the Caribbean | 4.27 | 0.41 | (3.46–5.09) | <0.001 |  |
| Mediterranean Basin | 3.27 | 0.72 | (1.86–4.68) | <0.001 |  |
| Middle East & North Africa | 3.22 | 0.45 | (2.34–4.10) | <0.001 |  |
| Nordic countries | 2.06 | 0.81 | (0.47–3.65) | 0.013 |  |
| South Asia | 2.14 | 0.62 | (0.92–3.36) | 0.001 |  |
| Sub-Saharan Africa | 3.13 | 0.45 | (2.24–4.01) | <0.001 |  |
| Female (% of total population) | -0.33 | 0.15 | (-0.62–-0.04) | 0.029 | 0.041 |
| GDP per capita (current US$) | 0.08 | 0.23 | (-0.37–0.53) | 0.728 | 0.001 |
| GHSI4: Health System | 0.58 | 0.16 | (0.27–0.89) | <0.001 | 0.104 |
| Hospital beds (per 1,000 people) | 0.29 | 0.16 | (-0.02–0.60) | 0.070 | 0.029 |
| Nurses (per 1,000 people) | 0.35 | 0.20 | (-0.05–0.74) | 0.089 | 0.025 |
| Out-of-pocket expenditure (% of current health expenditure) | 0.23 | 0.12 | (-0.01–0.46) | 0.059 | 0.031 |
| Over 65 years of age (%) | -0.18 | 0.27 | (-0.71–0.36) | 0.516 | 0.004 |
| Physicians (per 1,000 people) | -0.37 | 0.17 | (-0.70–-0.04) | 0.028 | 0.041 |
| Population density (P/Km²) | 0.31 | 0.11 | (0.09–0.53) | 0.006 | 0.064 |
| Unemployment (% of total labor force) | 0.23 | 0.11 | (0.01–0.45) | 0.045 | 0.035 |
| WASH: Sanitation | -0.35 | 0.24 | (-0.82–0.12) | 0.147 | 0.018 |
| WASH: Water | 0.85 | 0.22 | (0.42–1.29) | <0.001 | 0.114 |
| Adjusted $R^{2}$ | 0.658 |  |  |  |  |

**Supplementary Table S5.** Multiple linear regression analysis on log transformed COVID-19 case-fatality ratio for 136 countries, including countries in both northern and southern hemispheres. *β* beta coefficients, *SE* standard error, *95% CI* 95% confidence interval, *COVID-19 incidence* total confirmed cases of COVID-19 per one million population, *GDP* Gross Domestic Product, *GHSI* Global Health Security Index.

| **As of 14 September 2020 (N=136)** | **β** | **SE** | **(95% CI)** | **p-value** | **Partial** $\boldsymbol{R}^{\boldsymbol{2}}$ |
| --- | --- | --- | --- | --- | --- |
| Alcohol consumption (%) | -0.43 | 0.14 | (-0.71–-0.15) | 0.003 | 0.075 |
| COVID-19 incidence | -0.09 | 0.13 | (-0.33–0.16) | 0.477 | 0.004 |
| Ethnic region |  |  |  |  | 0.322 |
| East Asia | ref. |  |  |  |  |
| Europe (high income), North America & Oceania | 2.25 | 0.50 | (1.26–3.23) | <0.001 |  |
| Europe (low income), Russia & Central Asia | 2.26 | 0.44 | (1.40–3.13) | <0.001 |  |
| Latin America & the Caribbean | 3.05 | 0.46 | (2.16–3.95) | <0.001 |  |
| Mediterranean Basin | 2.24 | 0.60 | (1.07–3.42) | <0.001 |  |
| Middle East & North Africa | 2.50 | 0.52 | (1.49–3.52) | <0.001 |  |
| Nordic countries | 1.43 | 0.70 | (0.06–2.81) | 0.043 |  |
| South Asia | 0.79 | 0.57 | (-0.33–1.92) | 0.171 |  |
| Sub-Saharan Africa | 2.15 | 0.37 | (1.43–2.87) | <0.001 |  |
| Female (% of total population) | 0.01 | 0.14 | (-0.25–0.28) | 0.934 | <0.001 |
| GDP per capita (current US$) | -0.04 | 0.17 | (-0.37–0.28) | 0.793 | 0.001 |
| GHSI1: Prevention | 0.39 | 0.16 | (0.08–0.69) | 0.014 | 0.051 |
| GHSI5: Compliance | 0.14 | 0.12 | (-0.09–0.37) | 0.232 | 0.012 |
| International tourism, receipts (% of total exports) | -0.27 | 0.09 | (-0.44–-0.10) | 0.002 | 0.079 |
| Obesity prevalence (%) | -0.30 | 0.18 | (-0.65–0.04) | 0.088 | 0.025 |
| Over 65 years of age (%) | 0.80 | 0.21 | (0.37–1.22) | <0.001 | 0.107 |
| Physicians (per 1,000 people) | -0.34 | 0.15 | (-0.63–-0.05) | 0.023 | 0.045 |
| Population (N) | 0.26 | 0.09 | (0.08–0.45) | 0.006 | 0.065 |
| Population density (P/km²) | -0.21 | 0.10 | (-0.40–-0.02) | 0.030 | 0.041 |
| Unemployment (% of total labor force) | 0.18 | 0.11 | (-0.04–0.39) | 0.107 | 0.023 |
| Adjusted $R^{2}$ | 0.498 |  |  |  |  |

**Supplementary Table S6.** Multiple logistic regression analysis on dichotomized COVID-19 incidence by each ethnic region. *COVID-19 incidence* total confirmed cases of COVID-19 per one million population, *OR* odds ratios, *SE* standard error, *95% CI* 95% confidence interval, *COVID-19 test rate* number of COVID-19 tests performed per one million population, *GDP* Gross Domestic Product, *GHSI* Global Health Security Index, *WASH: Water* index that assesses the safety and accessibility to water, *WASH: Sanitation* index that assesses the facility sanitation.

| **As of 14 September 2020** | **OR** | **SE** | **(95% CI)** | **p-value** |
| --- | --- | --- | --- | --- |
| **Ethnic region: East Asia** | | | | |
| COVID-19 test rate | 8.69 | 2.68 | (0.14–20,573.36) | 0.420 |
| Female (% of total population) | 1.49 | 1.86 | (0.04–176.02) | 0.830 |
| Median age (years of age) | 0.23 | 1.74 | (0.00–4.37) | 0.397 |
| Nurses (per 1,000 people) | 605.46 | 3.63 | (4.48–25,230,921.31) | 0.078 |
| **Ethnic region: Europe (high income), North America & Oceania** | | | | |
| COVID-19 test rate | 1.52 | 1.64 | (0.05–52.63) | 0.798 |
| Female (% of total population) | 0.03 | 3.44 | (0.00–9.40) | 0.285 |
| GDP per capita (current US$) | 8.62 | 1.52 | (0.87–440.22) | 0.155 |
| GHSI3: Rapid Response | 0.94 | 0.78 | (0.17–4.63) | 0.934 |
| Median age (years of age) | 1,275.38 | 4.39 | (1.52–122,886,417.84) | 0.104 |
| **Ethnic region: Europe (low income), Russia & Central Asia** | | | | |
| Alcohol consumption (%) | 4.03 | 1.36 | (0.41–270.84) | 0.305 |
| COVID-19 test rate | 4.36 | 2.50 | (0.04–4,151.77) | 0.557 |
| Female (% of total population) | 0.19 | 3.85 | (0.00–968.08) | 0.663 |
| GDP per capita (current US$) | 0.88 | 6.79 | (0.00–399,357.78) | 0.985 |
| Hospital beds (per 1,000 people) | 1.13 | 1.48 | (0.04–34.57) | 0.935 |
| International tourism, receipts (% of total exports) | 0.41 | 0.91 | (0.03–1.91) | 0.324 |
| Median age (years of age) | 0.59 | 1.47 | (0.02–10.36) | 0.724 |
| **Ethnic region: Latin America & the Caribbean** | | | | |
| Female (% of total population) | 0.00 | 5.13 | (0.00–2.22) | 0.167 |
| Gini index | 6.67 | 1.47 | (0.53–230.68) | 0.196 |
| Median age (years of age) | 1.09 | 1.61 | (0.03–29.06) | 0.955 |
| Overall GHSI | 9.67 | 1.04 | (1.73–137.3) | 0.029 |
| **Ethnic region: Middle East & North Africa** | | | | |
| COVID-19 test rate | 404.41 | 4.10 | (0.91–30,412,938.92) | 0.143 |
| Female (% of total population) | 0.28 | 1.58 | (0.00–3.99) | 0.422 |
| GDP per capita (current US$) | 61.06 | 8.92 | (0.00–63,321,241,711.00) | 0.645 |
| GHSI4: Health System | 1.04 | 1.68 | (0.04–106.83) | 0.982 |
| Healthcare Access and Quality Index | 0.00 | 3.76 | (0.00–1.22) | 0.149 |
| WASH: Water | 56.47 | 3.20 | (0.85–457,085.81) | 0.208 |
| **Ethnic region: Sub-Saharan Africa** | | | | |
| Female (% of total population) | 1.54 | 3.65 | (0.00–2,665.86) | 0.906 |
| GDP per capita (current US$) | 15,284.52 | 16.19 | (0.00–∞) | 0.552 |
| Median age (years of age) | 0.05 | 5.46 | (0.00–1,616.69) | 0.585 |
| Physicians (per 1,000 people) | 1,254.15 | 7.59 | (0.20–∞) | 0.348 |
| Population (N) | 0.00 | 8.70 | (-∞–0.89) | 0.404 |
| Urban population (of total population) | 4.40 | 1.73 | (0.21–1,575.49) | 0.392 |
| WASH: Water | 40.47 | 2.35 | (1.79–112,889.29) | 0.115 |

**Supplementary Table S7.** Multiple logistic regression analysis on dichotomized COVID-19 case-fatality ratio by each ethnic region. *OR* odds ratios, *SE* standard error, *95% CI* 95% confidence interval, *COVID-19 incidence* total confirmed cases of COVID-19 per one million population, *COVID-19 test rate* number of COVID-19 tests performed per one million population, *GDP* Gross Domestic Product, *GHSI* Global Health Security Index, *WASH: Water* index that assesses the safety and accessibility to water, *WASH: Sanitation* index that assesses the facility sanitation.

| **As of 14 September 2020** | **OR** | **SE** | **(95% CI)** | **p-value** |
| --- | --- | --- | --- | --- |
| **Ethnic region: East Asia** | | | | |
| COVID-19 incidence | 0.02 | 2.65 | (0.00–1.29) | 0.133 |
| Education index | 2.36 | 1.78 | (0.04–125.94) | 0.629 |
| Female (% of total population) | 0.82 | 2.47 | (0.00–83.09) | 0.938 |
| GHSI4: Health System | 1.20 | 1.23 | (0.07–18.33) | 0.881 |
| Over 65 years of age (% of total population) | 32.08 | 2.68 | (0.67–107,688.91) | 0.196 |
| **Ethnic region: Europe (high income), North America & Oceania** | | | | |
| COVID-19 incidence | 0.85 | 1.06 | (0.06–7.05) | 0.874 |
| Female (% of total population) | 0.01 | 3.46 | (0.00–4.37) | 0.219 |
| GDP per capita (current US$) | 1.32 | 0.79 | (0.29–8.72) | 0.727 |
| GHSI1: Prevention | 4.16 | 1.80 | (0.10–195.10) | 0.427 |
| GHSI4: Health System | 0.35 | 1.78 | (0.01–10.45) | 0.554 |
| Median age (years of age) | 632.23 | 4.26 | (0.60–19,340,996.41) | 0.130 |
| Population density (P/km²) | 172.86 | 7.10 | (0.00–44,675,601,411.45) | 0.468 |
| Urban population (of total population) | 3.18 | 1.57 | (0.17–143.37) | 0.462 |
| **Ethnic region: Europe (low income), Russia & Central Asia** | | | | |
| COVID-19 incidence | 238,980,560,618.92 | 109,012.73 | (0–∞) | 1.000 |
| Female (% of total population) | 0.00 | 267,684.45 | (0–∞) | 0.999 |
| GDP per capita (current US$) | 0.00 | 330,756.82 | (0–∞) | 1.000 |
| Nurses (per 1,000 people) | 0.00 | 134,972.46 | (0–∞) | 1.000 |
| **Ethnic region: Latin America & the Caribbean** | | | | |
| Female (% of total population) | 56,988.93 | 6.15 | (3.38–496,462,000,000.00) | 0.075 |
| GDP per capita (current US$) | 0.00 | 4.07 | (0.00–0.25) | 0.049 |
| Hospital beds (per 1,000 people) | 0.04 | 2.26 | (0.00–0.93) | 0.150 |
| **Ethnic region: Middle East & North Africa** | | | | |
| COVID-19 incidence | 0.52 | 4.28 | (0.00–32,691,031.62) | 0.879 |
| Female (% of total population) | 10,121.42 | 10.89 | (17.01–∞) | 0.397 |
| GDP per capita (current US$) | 0.36 | 6.04 | (-∞–16,283.42) | 0.866 |
| Median age (years of age) | 0.11 | 2.50 | (0.00–1.67) | 0.382 |
| Population (N) | 19,935,847.47 | 19.97 | (0.50–∞) | 0.400 |
| Urban population (of total population) | 0.55 | 1.92 | (0.00–90.63) | 0.752 |
| **Ethnic region: Sub-Saharan Africa** | | | | |
| COVID-19 incidence | 9.72 | 2.62 | (0.12–10,930.29) | 0.386 |
| Education index | 9.98 | 1.29 | (1.06–202.47) | 0.074 |
| Female (% of total population) | 1.78 | 2.14 | (0.04–196.62) | 0.787 |
| GDP per capita (current US$) | 0.00 | 7.55 | (0.00–4.69) | 0.116 |
| Median age (years of age) | 0.18 | 2.69 | (0.00–41.82) | 0.518 |
| Overall GHSI | 0.91 | 1.00 | (0.12–7.15) | 0.928 |
